# Endothelial microparticles reduce ICAM-1 expression in a microRNA-222-dependent mechanism

**DOI:** 10.1111/jcmm.12607

**Published:** 2015-06-17

**Authors:** Felix Jansen, Xiaoyan Yang, Katharina Baumann, David Przybilla, Theresa Schmitz, Anna Flender, Kathrin Paul, Adil Alhusseiny, Georg Nickenig, Nikos Werner

**Affiliations:** a Department of Internal Medicine II, University Hospital Bonn, Rheinische Friedrich-Wilhelms UniversityBonn, Germany; b Feinberg Cardiovascular Research Institute, Northwestern University School of MedicineChicago, IL, USA

**Keywords:** endothelial microparticles, inflammation, microRNA-222, ICAM-1

## Abstract

Endothelial microparticles (EMP) are released from activated or apoptotic endothelial cells (ECs) and can be taken up by adjacent ECs, but their effect on vascular inflammation after engulfment is largely unknown. We sought to determine the role of EMP in EC inflammation. *In vitro*, EMP treatment significantly reduced tumour necrosis factor-α-induced endothelial intercellular adhesion molecule (ICAM)-1 expression on mRNA and protein level, whereas there was no effect on vascular cell adhesion molecule-1 expression. Reduced ICAM-1 expression after EMP treatment resulted in diminished monocyte adhesion *in vitro*. *In vivo*, systemic treatment of ApoE−/− mice with EMP significantly reduced murine endothelial ICAM-1 expression. To explore the underlying mechanisms, Taqman microRNA array was performed and microRNA (miR)-222 was identified as the strongest regulated miR between EMP and ECs. Following experiments demonstrated that miR-222 was transported into recipient ECs by EMP and functionally regulated expression of its target protein ICAM-1 *in vitro* and *in vivo*. After simulating diabetic conditions, EMP derived from glucose-treated ECs contained significantly lower amounts of miR-222 and showed reduced anti-inflammatory capacity *in vitro* and *in vivo*. Finally, circulating miR-222 level was diminished in patients with coronary artery disease (CAD) compared to patients without CAD. EMPs promote anti-inflammatory effects *in vitro* and *in vivo* by reducing endothelial ICAM-1 expression *via* the transfer of functional miR-222 into recipient cells. In pathological hyperglycaemic conditions, EMP-mediated miR-222-dependent anti-inflammatory effects are reduced.

## Introduction

Coronary artery disease (CAD) still represents the leading cause of mortality worldwide. The underlying disease, atherosclerosis, is initiated and propagated by a continuous damage of the vascular endothelium leading to the development of endothelial dysfunction and subsequent atherosclerotic lesion formation 1.

On a cellular level, endothelial dysfunction is based on progressive endothelial cell (EC) activation and apoptosis. In response, ECs release small membrane vesicles, termed endothelial microparticles (EMP). Endothelial microparticles are found in the plasma of healthy individuals, but are severely increased in individuals with vascular diseases such as hypertension or CADs. However, increasing evidence suggest that EMP are not only a surrogate marker for endothelial health or disease, but display vector function important for the intercellular exchange of biological information 2,3. Whether EMP might induce endothelial damage or promote vascular protection is still a matter of debate. Numerous data show that EMP can play a major role in inflammation, thrombosis and coagulation – all conditions involved in atherogenesis 4–6. In contrast, EMP were shown to promote cell survival, exert anti-inflammatory effects, counteract coagulation processes or induce endothelial regeneration 7,8, challenging the presumed deleterious role of EMP on vascular endothelial health. Recently, we demonstrated that EMP are taken up by endothelial target cells in an annexin I/PSR-dependent pathway, protect ECs against apoptosis and promote endothelial regeneration 9,10.

Here, we present evidence that EMP not only protect adjacent ECs from apoptosis but also promote anti-inflammatory effects by transferring microRNA (miR)-222 and thereby reducing intercellular adhesion molecule (ICAM)-1 expression on adjacent ECs.

## Materials and methods

### Cell culture and EMP generation

Human coronary artery endothelial cells (HCAEC, PromoCell, Heidelberg, Germany) were cultured in EC growth media with endothelial growth media SupplementMix (Promocell) under standard cell culture conditions (37°C, 5% CO_2_). Cells of passage 4–7 were used when 70–80% confluent. Endothelial microparticles were generated from HCAEC as previously described with minor changes 10. Briefly, confluent cells were starved by subjecting to basal media without growth media supplements for 24 hrs to induce apoptosis. After starvation, supernatant of apoptotic HCAEC was collected and centrifuged at 1500 × g for 15 min. to remove cell debris. The supernatant was centrifuged (20,000 × g, 40 min.) to pellet EMP. The obtained EMP were washed in sterile PBS (pH 7.4) and pelleted again at 20,000 × g. To generate EMP from ECs under hyperglycaemic conditions, confluent HCAEC were stimulated with 30 mM glucose for 72 hrs 11 and then subjected to basal media without growth media supplements for 24 hrs to generate EMP. Microparticles derived from glucose-treated ECs were defined as ‘injured’ EMP (iEMP). Pelleted EMP were resuspended in sterile PBS and used freshly. To determine the number of microparticles, TruCOUNT tubes (BD Biosciences, Heidelberg, Germany) were used. Endothelial microparticles were incubated with annexin V-FITC (BD Biosciences) in annexin V-binding buffer (10 mM HEPES, pH 7.4, 140 mM NaCl, 2.5 mM CaCl_2_). Annexin V-positive (AnnV^+^) EMP were enumerated by flow cytometry. Endothelial microparticle concentrations were calculated by the following formula: (number of events for annexin V/number of events in TruCOUNT bead region) × (number of TruCOUNT beads per test/test volume). Endothelial microparticles were used at the concentration of 2000 AnnV^+^ MP/μl for all experiments unless indicated otherwise.

For *in vitro* tumour necrosis factor (TNF)-α experiments, confluent HCAEC were pre-treated with EMP for 1 hr and then subjected to TNF-α (20 ng/ml; R&D Systems, Wiesbaden, Germany) for 24 hrs. For oxidized LDL (oxLDL) experiments, confluent HCAEC were pre-treated with EMP for 1 hr and then subjected to oxLDL (20 μg/ml) for 24 hrs as previously described 12.

Endothelial microparticles uptake experiments were performed as previously described 9. In brief, EMP were incubated for 30 min. with 1 μM fluorescent calcein AM (Invitrogen Life Technologies, Darmstadt, Germany) at 37°C, washed and centrifuged twice at 20,000 × g, and resuspended in sterile PBS. Human coronary artery endothelial cells were incubated with calcein acetoxymethyl (AM)-labelled EMP for different time frames. After three washing steps, HCAEC were fixed in 4% paraformaldehyde, blocked with 5% bovine serum albumin, and nuclei were stained with DAPI (Vector Laboratories, Peterborough, UK). Zeiss Axiovert 200M microscope (Carl Zeiss AG, Oberkochen, Germany) and AxioVision Software (Carl Zeiss AG, Oberkochen, Germany) were used to visualize the uptake of EMP.

### Animals and procedures

All animal experiments were performed in accordance with the Directive 2010/63/EU of the European Parliament. All animal work was approved and supervised by the regulatory authority of the state of Nordrhein Westfalen and in compliance with German animal protection laws.

For this study, 10- to 12-week-old apolipoprotein E-deficient mice (ApoE−/−, C57BL/6J genetic background) from Charles River were used. All animals were kept in accordance with standard animal care requirements and maintained in a 22°C room with a 12-hr light/dark cycle, and received food and drinking water *ad libitum*. All mice received a high-fat, cholesterol-rich diet that contained 21% fat, 19.5% casein and 1.25% cholesterol (Ssniff special nutrition, Soest, Germany) for a total of 8 weeks (or 4 weeks for EMP^miR-222-down-regulated^ experiments) and were injected intravenously twice per week with 1 × 10^7^ AnnexinV^+^ EMP diluted in 200 μl sterilized PBS. Afterwards, the mice were killed under ketaminehydrochloride (300 mg/kg, Ketanest; Pharmacia, Berlin, Germany)–xylazinehydrochloride (30 mg/kg, Rompun 2%; Bayer, Leverkusen, Germany) anaesthesia. Respiration rate, muscle relaxation and different reflexes were used to indicate the adequacy of anaesthesia. Aortic segments were collected and processed immediately after sacrifice.

### Analysis of ICAM-1 expression in descending aorta

Descending aortas were embedded in Tissue Tek OCT embedding medium (Tissue Tek O.C.T. embedding medium, Sakura, Alphen aan den Rijn, Netherlands), snap-frozen and stored at −80°C. Samples were sectioned on a Leica Biosystems (Wetzlar, Germany) cryostat (6 μm) and were placed on slides. Descending aorta sections were stained with anti-ICAM-1 (goat antimouse antibody, R&D Systems) and anti-CD31 (rat antimouse antibody, Santa Cruz Biotechnology). Nuclei were revealed with DAPI (Vector Laboratories, Santa Cruz Biotechnology, Heidelberg, Germany). All sections were examined under a Zeiss Axiovert 200M microscope using AxioVision version 4.5.0 software.

### Western blot

Cells were lysed in RIPA buffer (150 mM NaCl, 1.0% Nonidet P-40, 0.5% deoxycholate, 0.1% SDS, and 50 mM Tris, pH 8.0) containing 1 mM Na_3_VO_4_, 5 mM NaF and protease inhibitor cocktail (Roche, Grenzach-Wyhlen, Germany) at 4°C. Protein concentration was measured using Lowry protein assay (Bio Rad, Munich, Germany). Equal amounts of proteins (25 μg) were loaded into 12% SDS electrophoresis and transferred onto polyvinylidene difluoride (PVDF) membranes. Blots were incubated with the appropriate primary antibodies (anti-ICAM-1, Santa Cruz Biotechnology; anti-Glyceraldehyde 3-phosphate dehydrogenase (GAPDH), Hytest), followed by the correspondent HRP-conjugated secondary antibodies and proteins were revealed by chemiluminescence using the ECL kit (GE healthcare, Chalfont St. Giles, Buckinghamshire, UK). GAPDH was used as the loading control.

### Real-time PCR

Human coronary artery endothelial cells were lysed in Trizol (Invitrogen). RNA was isolated according to the manufacturer’s instructions and quantified using Nanodrop spectrophotometer. Then, 1 μg of the isolated total RNA was reversely transcribed using Omniscript RT Kit (Qiagen, Hilden, Germany) according to the manufacturer’s protocol. The single-stranded cDNA was amplified by real-time polymerase chain reaction (real-time PCR) with the TaqMan system [ABI-7500 (Life technologies, Darmstadt, Germany) fast PCR System] using SYBR-Green dye. Primers used: ICAM-1 forward 5′-CGCAAGGTGACCGTGAATGT, reverse 5′-CGTGGCTTGTGTGTTCGGTT; vascular cell adhesion molecule (VCAM)-1 forward 5′-AGTCAGGAATTTCTGGAGGATGC, reverse 5′-GCAGCTTTGTGGATGGATTCAC; 18s forward GTAACCCGTTGAACCCCATT, reverse CCATCCAATCGGTAGTAGCG. mRNA expression was normalized to endogenous 18s rRNA.

### microRNA expression in microparticles and HCAEC

Total RNA is isolated out of EMP, iEMP and HCAEC by Trizol (Invitrogen) extraction method according to instruction of the manufacturer. To increase the yield of small RNAs, the RNA is precipitated in ethanol at −20°C overnight with glycogen (Invitrogen). RNA is quantified using Nanodrop spectrophotometer. Then, 10 ng of the total RNA was reversely transcribed using TaqMan® microRNA reverse transcription kit (Applied Biosystems, Life technologies, Darmstadt, Germany) according to the manufacturer’s protocol. Taqman miRNA assays (Applied Biosystems) were used to measure miR-222 levels on a 7500 HT Real-Time PCR machine (Applied Biosystems). RNU-6 was used as an endogenous control. Delta Ct method was used to quantify relative miR expression.

### Taqman microRNA array

RNA (more than 500 ng) isolated from EMP and HCAEC was converted to cDNA by priming with a mixture of looped primers (Human Mega Plex Primer Pools; Applied Biosystems). miR profiles in EMP and HCAEC were performed with TaqMan® Array MicroRNA Cards (Card A; Applied Biosystems) for a total of 384 unique assays specific to human miRNAs under standard real-time PCR conditions. PCR was carried out on an Applied Biosystems 7900HT thermocycler using the manufacturer’s recommended programme. Detailed analysis of the results was performed with the DataAnalysis v3.0 Software (Applied Biosystems). CT values above 35 were defined as undetectable.

### Transfection of HCAEC

To generate EMP^miR-39^, EMP^miR-222-down-regulated^ and EMP^mock-transfected^, HCAEC were transfected with cel-miR-39 (1 nM; Qiagen), miR-222 inhibitor or microRNA inhibitor control (1 nM, all from Applied Biosystems) using lipofectamine 2000 (Invitrogen) for 16 hrs and exposed to media without growth media supplements for 24 hrs to generate modified EMP as previously described 10.

### Monocyte adhesion assay

THP-1 cells were labelled with calcein AM (10 μM; Sigma-Aldrich, St. Louis, Missouri, USA). Confluent HCAEC were incubated with 1 × 10^5^/ml calcein AM-labelled THP-1 cells for 30 min. Unbound cells were removed by gentle washing, and nuclei were stained with DAPI (Vector Laboratories). The number of adherent monocytes was analysed using immunofluorescence microscope (Zeiss Axiovert 200M microscope) by counting nine random 100× fields in each sample.

### Study participants of the clinical study

Between August 2012 and July 2013, 74 patients undergoing coronary angiography were screened for inclusion of stable CAD. Six patients with clinical presentation of acute or subacute myocardial infarction were excluded from the study. Patients with malignant, inflammatory diseases, or severe hepatic or renal dysfunction were also excluded from the study. Informed consent was obtained from all patients and the ethics committee of the University of Bonn approved the study protocol.

### Coronary angiography

Cardiac catheterization was performed according to the guidelines for coronary angiography of the American College of Cardiology and the American Heart Association. The extent of CAD was scored, by at least two independent interventional cardiologists. Angiographic CAD was defined as stenosis of 50% in at least one major epicardial coronary artery. Biplane ventriculography was performed in standard projections. The ejection fraction was calculated by dividing the end-diastolic and end-systolic LV areas with an automated computer system (Digital Cardiac Imaging Software; Philips, Amsterdam, Netherlands).

### Preparation of blood samples

Venous blood samples were collected from patients in EDTA blood tubes (Sarstedt, Monovette EDTA K; Sarstedt AG, Germany). Additional blood samples for routine analyses were obtained. Blood was drawn prior to heparin application, so there was no confounding effect of heparin on miR analysis.

Blood was centrifuged at 1500 × g for 15 min. followed by centrifugation at 13,000 × g for 2 min. to generate platelet-deficient plasma. The deprived plasma samples were immediately stored in −80°C until miR levels were analysed.

### RNA isolation

RNA was isolated from plasma by using TRIzol-based RNA isolation protocol. For each patient, 250 μl total plasma was diluted in 750 μl TRIzol® LS (Life technologies, Darmstadt, Germany) to measure plasma microRNA levels. *Caenorhabditis elegans* miR-39 (cel-miR-39, 5 nM; Qiagen) was spiked in TRIzol for normalization of miR content as described 13,14. To increase the yield of small RNAs, the RNA was precipitated in ethanol at −20°C overnight with glycogen (Invitrogen).

### Quantification of miRs by quantitative PCR

RNA was quantified using Nanodrop spectrophotometer. Exactly, 10 ng of the total RNA was reversely transcribed using TaqMan® microRNA reverse transcription kit (Applied Biosystems) according to the manufacturer’s protocol. miR-222 in plasma was detected by using Taqman miRs assays (Applied Biosystems) on a 7500 HT Real-Time PCR machine (Applied Biosystems). Cel-miR-39 was used as an endogenous control. For all miRs, a Ct value above 40 was defined as undetectable. 2^−ddCT^ method was used to quantify relative microRNA expression. Values were normalized to cel-miR-39.

### Stimulation of target HCAEC with patients’ serum

Sera collected from 10 randomly chosen participants with or without CAD were used for *ex vivo* stimulation of HCAEC as previously described 15. Briefly, HCAEC were incubated for 24 hrs (37°C) with 10% of the conditioned serum samples (900 μl growth factor-deprived medium + 100 μl of serum). Afterwards, serum was removed and the cells were washed three times with sterile PBS. Intercellular adhesion molecule-1 and miR-222 expression was analysed using real-time PCR as described in the previous sections.

### Statistical analysis

Data are expressed as mean ± SEM. Means between two categories were compared with the use of a two-tailed, unpaired Student’s *t*-test. The one-way anova test was used for comparisons of categorical variables. For *post hoc* analysis, the Bonferroni test was applied. Mann–Whitney *U*-test was used to analyse variables with skewed distribution. Statistical significance was assumed when a null hypothesis could be rejected at *P* < 0.05. Statistical analysis was performed with GraphPad Prism 5.

## Results

### EMP promote anti-inflammatory effects

**Figure 1:**
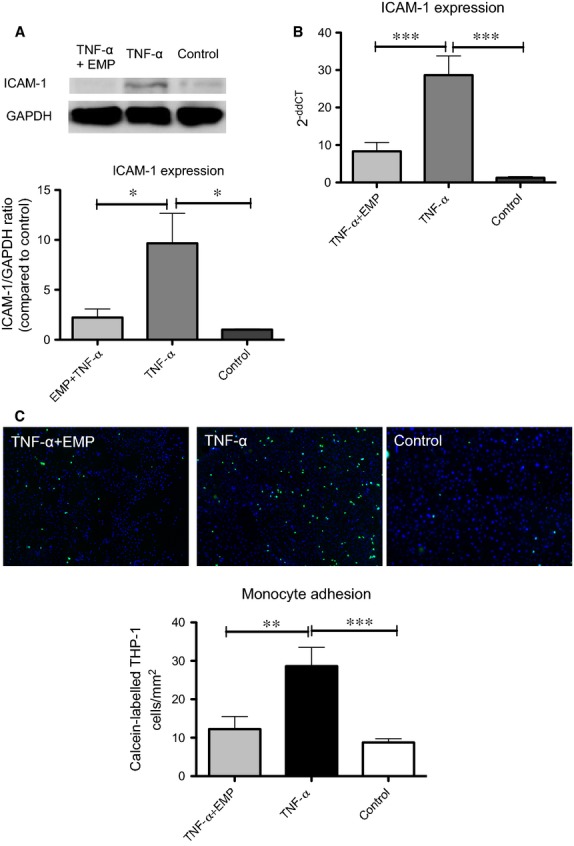
EMP decrease TNF-α-induced ICAM-1 expression on HCAEC and monocyte adhesion to HCAEC. HCAEC were pre-treated with EMP or vehicle for 1 hr and then stimulated with TNF-α or vehicle for 24 hrs. (A) ICAM-1 expression on HCAEC was detected by Western blot. GAPDH was used as endogenous control, *P < 0.05, *n* = 4. (B) ICAM-1 mRNA expression was analysed by real-time RT-PCR. 18s was used as endogenous control, ***P < 0.001, *n* = 8–10. (C) HCAEC were incubated with 1 × 10^5^/ml calcein AM-labelled THP-1 cells for 30 min. Unbound cells were removed by gentle washing, and nuclei were stained with DAPI (blue). The number of adherent monocytes (green) was analysed using immunofluorescence microscope by counting nine random 100 ×  fields in each sample. Results are representative of at least three independent experiments, **P < 0.01, ***P < 0.001, *n* = 8–10.

**Figure 2:**
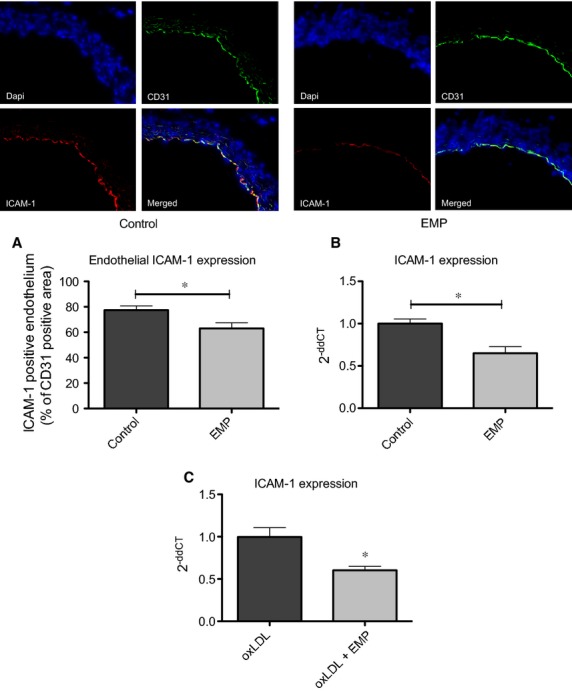
EMP reduce vascular endothelial ICAM-1 expression. ApoE−/− mice, 10- to 12-week old, receiving cholesterol-rich high-fat diet were treated twice per week with 1 × 10^7^ AnnV+ EMP or vehicle as control over a period of 8 weeks. (A) ICAM-1 mRNA levels of murine aorta were analysed by real-time PCR. 18s was used as endogenous control. (B) ICAM-1 (red) and CD31 (green) expression in the descending aorta was assessed by immunofluorescence. Nuclei were stained with DAPI (blue). Original magnification: 100 × . Data were expressed as a per cent of CD31^+^ area, *P < 0.05, *n* = 8. (C) ICAM-1 mRNA levels of oxLDL-stimulated target cells (20 μg/ml) were analysed by real-time PCR. 18s was used as endogenous control.

As vascular inflammation is one of the key elements in the development of vascular diseases, we first investigated how EMP, derived from activated ECs, influence the expression of adhesion proteins ICAM-1 and VCAM-1 on adjacent ECs. Expression of adhesion proteins treated with or without EMP was induced using TNF-α. Treatment of HCAEC with EMP significantly reduced TNF-α-induced expression of ICAM-1 on a protein and mRNA level, whereas VCAM-1 expression was not affected (Fig.1A and B, Fig. S1). Stimulation of target HCAEC with EMP revealed a time-dependent uptake of EMP (Fig. S2). Next, we evaluated if down-regulation of ICAM-1 might functionally affect monocyte adhesion to ECs. Therefore, calcein AM-labelled monocytes were co-incubated with TNF-α-stimulated HCAEC, which had been pre-treated with EMP or vehicle as control for 24 hrs. Endothelial microparticle treatment was associated with decreased monocyte adhesion to HCAEC compared to vehicle treatment (12.27 ± 3.2 *versus* 28.61 ± 4.9, *P* < 0.01, *n* = 8–10, Fig.1C). These data indicate that EMP-induced down-regulation of ICAM-1 might be functionally relevant in terms of reduced monocyte adhesion. Recently, we could demonstrate that human EMP are taken up by murine ECs *in vivo* and influence murine endothelial function 10. We therefore assessed whether human EMP might also affect endothelial ICAM-1 expression in ApoE−/− mice fed high cholesterol diet over a period of 8 weeks. In accordance with our *in vitro* findings, EMP significantly reduced murine vascular endothelial ICAM-1 expression, whereas VCAM-1 was unaffected (Fig.2A and B, Fig. S3). In parallel to the *in vivo* model, the anti-inflammatory effect of EMP was confirmed in an additional *in vitro* model, where EMP treatment significantly reduced oxLDL-induced ICAM-1 expression (1.0 ± 0.1 *versus* 0.6 ± 0.04, *P* < 0.05, *n* = 4–5).

### EMP-mediated ICAM-1 down-regulation is miR-222 dependent

**Figure 3:**
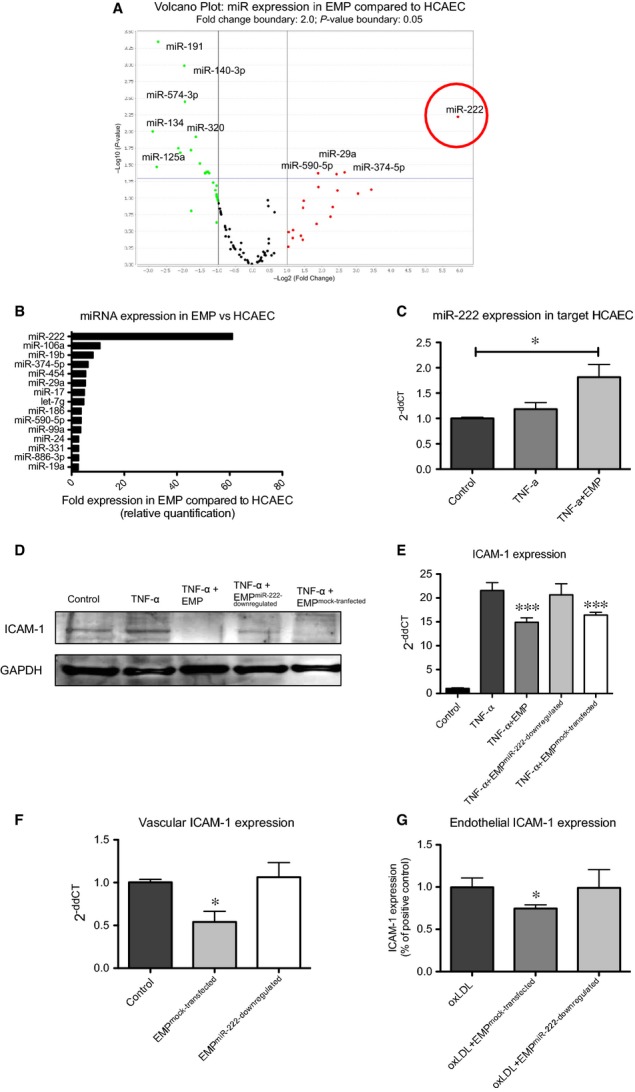
EMP contain and transfer functional miR-222 to endothelial target cells. (A) Volcano plot of microRNA profile in EMP and HCAEC as determined *via* TaqMan miRNA array (*n* = 3). Right vertical line represents the border for twofold change in miR expression [points on the right side of that line symbolize miRs that are >2 ×  higher expressed in EMP *versus* HCAEC (log2)]. Horizontal line displays the border for significant regulation. Points above that line represent significantly regulated miRs (*P* < 0.05(−log10)). (B) Top 15 miRs higher expressed in EMP compared to HCAEC. RNU6 was used as endogenous control. (C) Expression of miR-222 in HCAEC detected by Taqman microRNA assay, RNU6 was used as endogenous control, *P < 0.05, *n* = 6–8. (D and E) EMP^miR-222-down-regulated^ were derived from miR-222 inhibitor-transfected parent cells. HCAEC pre-treated with EMP, EMP^miR-222-down-regulated^, EMP^mock-transfected^ or vehicle were stimulated with TNF-α or vehicle for 24 hrs. (D) ICAM-1 expression was analysed by Western blot. (E) ICAM-1 mRNA levels were analysed by real-time PCR. 18s was used as endogenous control, ***P < 0.001, *n* = 8. (F) ICAM-1 mRNA levels from ascending aortas from ApoE−/− mice were analysed by real-time PCR. 18s was used as endogenous control, *P < 0.5, *n* = 8–10. (G) ICAM-1 mRNA levels from HCAEC treated with 20 μg/ml oxLDL and with or without EMP were analysed by real-time PCR. 18s was used as endogenous control, *P < 0.05 *versus* oxLDL and EMP^miR-222-down-regulated^, *n* = 8.

Increasing evidence suggests that MP emerge as major transport vehicles for miRs and that the effects of MP depend on the MP-containing miR expression 16–18. To investigate the potential pathway how EMP reduce endothelial ICAM-1 expression, TaqMan microRNA array analysis was performed to measure the expression of 384 different miR in EMP and HCAEC. Among those, miR-222 was the strongest regulated miR between EMP and HCAEC with a significantly higher expression in EMP (Fig.3A and B). To investigate whether EMP can effectively transfer miRs into target cells, we first performed the following control experiments. HCAEC were transfected with cel-miR-39, which is naturally expressed in *C.elegans*, and microparticles were generated from these cells as described. cel-miR-39-containing EMP (EMP^miR-39^) were then incubated with HCAEC and subsequent PCR analysis revealed significant amounts of cel-miR-39 in HCAEC, indicating that microparticles can indeed transfer miRs to target cells (CT value = 26 *versus* non-detectable, *n* = 6, *P* < 0.05). Extending these experiments, treatment of HCAEC with EMP markedly increased the abundance of miR-222 in target ECs, whereas TNF-α treatment alone did not influence miR-222 level (1 ± 0.0 *versus* 1.18 ± 0.1 *versus* 1.81 ± 0.25, *P < 0.05, *n* = 6–8, *P* < 0.05, Fig.3C). These data demonstrate an efficient transfer of miR-222 by EMP to recipient cells. Next, three online databases (miRanda, PicTar5, TargetScanHuman6.0) were independently used to find predicted mRNA targets for miR-222. Interestingly, all of the used databases predicted ICAM-1 as target for miR-222.

To further confirm ICAM-1 as target for miR-222 in EMP, miR-222 inhibitor was used to generate EMP^miR-222-down-regulated^ and corresponding EMP^mock-transfected^. Treatment of endothelial target cells with the modified EMP showed that EMP contain functional miR-222 that regulate ICAM-1 expression in target ECs (***P < 0.01, *n* = 5–6, Fig.3D and E). Down-regulation of miR-222 in EMP abrogated EMP-induced inhibition of ICAM-1 expression after TNF-α stimulation. Treatment of ApoE−/− mice fed high cholesterol diet with EMP^miR-222-down-regulated^ abrogated the EMP-promoted inhibition of vascular ICAM-1 expression (*P < 0.05, *n* = 7–8, Fig.3F). Finally, in parallel to the *in vivo* model, stimulation of oxLDL-treated HCAEC with EMP^miR-222-down-regulated^ abolished the EMP-induced inhibition of endothelial ICAM-1 expression (*P < 0.05, *n* = 6–7, Fig.3G).

Efficient miR-222 down-regulation in EMP using miR-222 inhibitor was confirmed by real-time PCR (Fig. S4).

### EMP derived from glucose-treated ECs contain less miR-222 and lose their anti-inflammatory capacity

**Figure 4:**
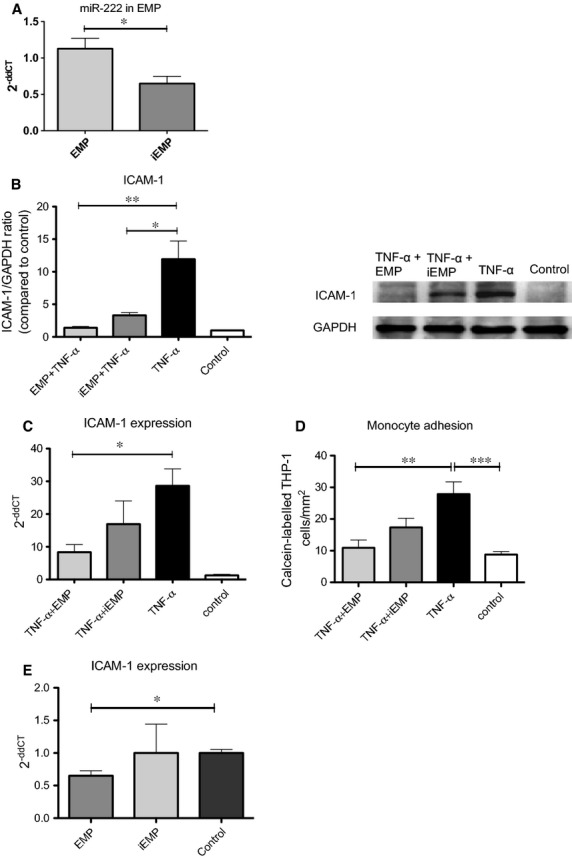
High glucose condition reduces miR-222 in iEMP, which subsequently lose their anti-inflammatory properties. (A) TaqMan real-time PCR analysis of miR-222 expression in EMP and iEMP. RNU6 was used as endogenous control (*P < 0.05, *n* = 8). (B and C) HCAEC pre-treated with EMP, iEMP or vehicle as control were stimulated with TNF-α for 24 hrs. (B) ICAM-1 expression was analysed by Western blot, *P < 0.05, **P < 0.01, *n* = 4. (C) ICAM-1 mRNA levels were analysed by real-time PCR. 18s was used as endogenous control, *P < 0.05, *n* = 8. (D) Monocyte adhesion *in vitro* was assessed as described in Figure1, ***P < 0.001. (E and F) ApoE−/− mice, 10- to 12-week old, receiving cholesterol-rich high-fat diet were treated twice per week with 1 × 10^7^ AnnV+ EMP, iEMP or vehicle over a period of 8 weeks. ICAM-1 mRNA levels of murine aorta were analysed by real-time PCR. 18s was used as endogenous control.

Recently, we could demonstrate that the content and subsequent effect of endothelial MP depends on the state of the releasing cell 10. As EMP are increased in diabetic patients and potentially linked to vascular complications in diabetes 19,20, we sought to determine whether miR content and effects of EMP derived under high glucose concentration might differ from EMP derived from untreated cells. Endothelial microparticles generated from glucose-treated cells were defined as iEMP. Real-time PCR experiments revealed a significantly reduced miR-222 expression in iEMP compared to EMP (0.64 ± 0.09 *versus* 1.12 ± 0.14, *n* = 14–16, *P* < 0.05, Fig.4A).

As we found that genetic knock-down of miR-222 in EMP abrogated EMP-mediated inhibition of ICAM-1 expression (Fig.3D and E), we next tested the effect of a biological reduction in miR-222 expression in iEMP derived under hyperglycaemic conditions.

Importantly, reduced miR-222 level in iEMP resulted in altered inhibition of TNF-α-induced endothelial ICAM-1 expression (Fig.4B and C) and subsequent monocyte adhesion (Fig.4D). *In vivo*, EMP-mediated inhibition of vascular ICAM-1 expression from ApoE−/− mice was significantly impaired after iEMP treatment compared to EMP (Fig.4E). Apparently, under pro-inflammatory conditions, iEMP derived under pathological hyperglycaemic concentrations lose their anti-inflammatory properties because of decreased miR-222 levels. Based on these data, one may speculate that high glucose conditions not only directly harm the endothelium but also regulate miR expression in EC-derived microparticles, subsequently attenuating anti-inflammatory processes.

### Coronary artery disease is associated with reduced circulating miR-222 levels

**Figure 5:**
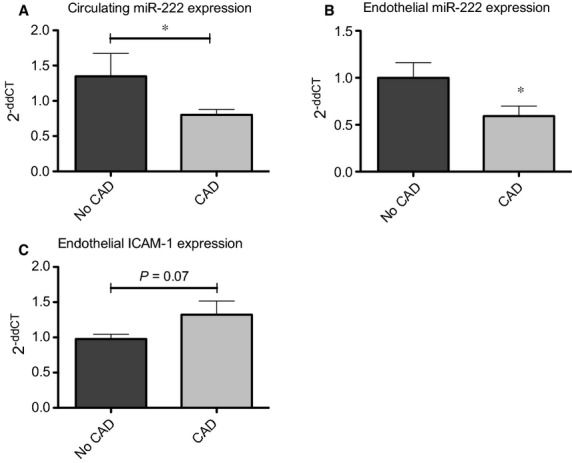
Coronary artery disease is associated with reduced circulating miR-222 levels. (A) miR-222 expression was significantly reduced in patients with coronary artery disease. The 2^−ddCT^ method was used to quantify relative microRNA expression compared to control group (No CAD). cel-miR-39 was used as internal control. (B and C) miR-222 and ICAM-1 expression in serum-treated HCAEC was analysed by real-time PCR, *P < 0.05.

**Figure 6:**
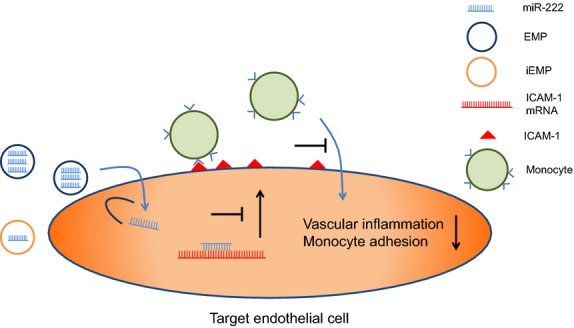
Proposed mechanism: Endothelial microparticles reduce ICAM-1 expression in a microRNA-222-dependent mechanism.

As we found that miR-222 selectively inhibits endothelial ICAM-1 expression, which contributes to vascular inflammation and atherosclerosis, we finally explored whether miR-222 levels are altered in patients with CAD (CAD). In a total of 68 patients presenting with symptoms of stable CAD, levels of circulating miR-222 were analysed (baseline characteristics are shown in Table1). In patients with angiographically proven CAD, miR-222 levels were significantly reduced compared to patients without CAD (1.00 ± 0.13 *versus* 0.7 ± 0.05, *P* < 0.05, Fig.5A). To explore the effect of patient’s sera on the endothelium, HCAEC were stimulated with sera from patients with and without CAD for 24 hrs, and miR-222 and ICAM-1 expression in target cells was assessed. Stimulation of HCAEC with sera from CAD patients resulted in a significantly reduced miR-222 expression compared to sera from patients without CAD (1.00 ± 0.16 *versus* 0.59 ± 0.14, *P < 0.05, *n* = 10, Fig.5B). Furthermore, ICAM-1 showed a clear trend towards an augmented expression in HCAEC treated with sera from CAD patients (1.00 ± 0.06 *versus* 1.32 ± 0.19, *P* = 0.07, *n* = 10, Fig.5C), supporting our hypothesis that miR-222 inhibits ICAM-1 expression (Fig.6).

**Table 1:** Baseline characteristics

	No CAD (*n* = 20)	Stable CAD (*n* = 60)	Total (*n* = 80)	*P*-value
Age, years	66.55 ± 9.5	64.9 ± 10.4	65.33 ± 10.2	0.538
Gender, no. (%)				
Female	13 (65.0%)	10 (16.7%)	23 (28.8%)	
Male	7 (35.0%)	50 (83.3%)	57 (71.3%)	
Cardiovascular risk factors, no. (%)				
Arterial hypertension	16 (80.0%)	49 (81.6%)	65 (81.2%)	0.761
Hyperlipoproteinemia	7 (35%)	40 (66.7%)	47 (58.7%)	0.016
Diabetes	5 (25%)	21 (35.0%)	26 (32.5%)	0.489
Family history of CAD	3 (15%)	14 (23.3%)	17 (21.2%)	0.472
Smoking	4 (20%)	20 (33.3%)	24 (30%)	0.269
Body mass index, kg/m^2^	29.0 ± 7.8	28.8 ± 4.4	28.8 ± 5.4	0.963
Laboratory parameters				
Cholesterol (mg/dl)	192 ± 46.3	174.4 ± 42.0	178.8 ± 43.5	0.119
LDL cholesterol (mg/dl)	114.7 ± 35.6	105.6 ± 32.6	107.9 ± 33.4	0.298
HDL cholesterol (mg/dl)	57.8 ± 18.7	42.0 ± 10.5	46.0 ± 14.6	0.02
Triglycerides (mg/dl)	119.7 ± 51.3	195.1 ± 293.4	176.2 ± 256.9	0.258
Serum creatinine (mg/dl)	0.85 ± 0.23	1.01 ± 0.27	0.97 ± 0.27	0.015
Glomerular filtration rate (ml/min)	67.5 ± 7.3	66.1 ± 10.4	65.5 ± 9.7	0.578
Leucocytes (10^9^/l)	6.99 ± 1.61	7.80 ± 2.24	7.61 ± 2.12	0.127
C-reactive protein (mg/l)	3.47 ± 3.09	4.74 ± 9.12	4.42 ± 8.03	0.545
Medical history, no. (%)				
Previous MI (6 months)	0	25 (41.7)	25 (31.3)	
Previous bypass	0	13 (21.7)	13 (16.3)	
Previous PCI	0	48 (80.0)	48 (60)	
Medication on admission, no. (%)				
ACE inhibitors	6 (30)	45 (75.0)	51 (63.8)	0.000192
Angiotensin receptor blockers	8 (40)	11 (18.3)	19 (23.8)	0.09
Beta-blockers	14 (70)	53 (88.3)	67 (83.8)	0.118
Calcium channel blockers	7 (35)	13 (21.7)	20 (25.0)	0.238
Diuretics	13 (65)	25 (41.7)	38 (47.5)	0.072
Statins	7 (35)	57 (95.0)	64 (80.0)	0.00027
Nitrates	0	2 (3.3)	2 (2.5)	0.415
Aspirin	1 (5)	56 (93.3)	57 (71.3)	0
Clopidogrel	0	21 (35.0)	21 (26.3)	10^−6^

CAD: coronary artery disease; LDL: low-density lipoprotein; HDL: high-density lipoprotein; MI: myocardial infarction; PCI: percutaneous coronary intervention; ACE: angiotensin-converting enzyme.

## Discussion

Endothelial cell activation and apoptosis, both key elements in the development of vascular disease, led to the release of EMP. It has been widely speculated that microparticle release from apoptotic ECs may protect adjacent cells from cell death and promote vasculoprotection 21,22. Indeed, uptake of EMP in ECs leads to abrogation of camptothecin-induced apoptosis 23. Based on these findings, we hypothesized that EMP not only inhibit apoptosis but also modulate inflammation.

In this study, we found that EMP reduce TNF-α-induced endothelial inflammation *in vitro* by transferring functional miR-222 to target ECs with subsequent down-regulation of ICAM-1. These findings emphasize that EMP act as anti-inflammatory mediator by functionally influencing endothelial target cells.

What might be the potential underlying biological concept of these observations? Evidence support the notion that EMP are increased in all conditions of systemic EC damage, such as cardiovascular diseases as well as autoimmune diseases. In this context, previous publications reported that EMP may induce thrombosis, inflammation, apoptosis and endothelial dysfunction, pinpointing towards the impression that EMP are deleterious in vascular homoeostasis 24,25. Interestingly, EMP have additional biological vector function, which may shed a different light on their role in pathologies. We recently demonstrated that EMP protect target ECs from camptothecin-induced apoptosis through inhibition of p38 activation and restoration of MKP-1 expression. Another study revealed that apoptotic bodies derived from apoptotic ECs can transfer miR-126 to target cells, inducing CXCL12-dependent vascular protection *in vitro* and *in vivo* 22. Based on these data, one may speculate that apoptosis of ECs with concomitant release of MP is an important feedback mechanism, protecting adjacent ECs against further cell damage.

Microparticles can transfer proteins, cytokines, mRNA or miR to target cells and influence their biological behaviour 26. Accordingly, the role of EMP has changed from being only a marker of vascular integrity towards a relevant effector in intercellular signalling 27. Importantly, very recent data suggest that MP represent major transport vehicles for miRs and that the effects of MP depend on the MP-containing miR expression 16,17. In this context, it has been shown that monocytic-derived MPs transfer functional miR-150 to ECs and promote EC migration by targeting c-Myb 28. Furthermore, Hergenreider *et al*. recently described an atheroprotective communication between ECs and smooth muscle cells through miRNAs 143/145 29.

Our miR array experiments show that miR-222 is the highest regulated miR between EMP and their releasing endothelial parent cells. Further experiments demonstrated increased miR-222 level in EMP-treated endothelial target cells, suggesting an efficient transfer of miR-222 by EMP. Intercellular adhesion molecule-1 is one predicted target of miR-222 and a direct inhibition of ICAM-1 by miR-222 has been demonstrated by Ueda *et al*. 30. We found that EMP contain and transfer miR-222 into endothelial target cells and inhibit ICAM-1 expression. Gain and loss of function experiments showed that knock-down of miR-222 in EMP abrogated the EMP-mediated anti-inflammatory effects. These results indicate that EMP can act as biological vectors that deliver functional miR-222 into recipient ECs (Fig.6).

The role of miR-222 in vascular biology is just beginning to become apparent. In vascular smooth muscle cells (VSMCs), miR-222 has been shown to act as crucial modulator of proliferation by targeting p27^Kip1^ and p57^Kip2^ 31. Furthermore, miR-222 controls neovascularization by regulating signal transducer and activator of transcription 5A expression 32. Interestingly, the effects of miR-222 seem to be cell specific with opposite effects on VSMCs and ECs 33.

According to our miR array data, apoptotic ECs seem to specifically package miR-222 in EMP. Interestingly, others found that ECs exposed to inflammatory stimuli showed reduced levels of miR-222 compared to untreated ECs 32. Assuming an anti-inflammatory role of miR-222, one may speculate that apoptotic EC package miR-222 as a vasculo-protective message into EMP to shelter adjacent ECs from further damage.

However, evidence suggest that the effect of EMP on target cells depends on the condition of the releasing cell 34. We found that iEMP derived under high glucose conditions show significantly reduced miR-222 expression and reduced anti-inflammatory capacity *in vitro* compared to EMP derived from untreated cells. These data indicate that EMP released from damaged ECs lose their potential to protect adjacent ECs from TNF-α-induced inflammation, and difference in miR expression might be one possible mechanism. The clinical relevance of high glucose conditions on miR expression has already been demonstrated by Zampetaki *et al*., who found that diabetic patients and apoptotic bodies derived under hyperglycaemic conditions show reduced miR-126 expression compared to healthy controls 35. Endothelial cells in general are very responsive to glucose levels. Hyperglycaemic conditions promote formation of multiple biochemical species such as advanced glycation end products, reactive oxygen species (ROS) and nitrosylated species, which subsequently contribute to the pro-inflammatory and pro-adhesive phenotypic changes of vascular ECs under hyperglycaemic conditions. Interestingly, accumulating evidence suggests that ROS production regulates microRNA expression. Reactive oxygen species production up-regulates miR-21 in VSMCs and increases miR-210 in adipose-derived stem cells 36. Li *et al*. demonstrated that insulin-induced ROS production down-regulates miR-145 and miR-128 36. Extending these findings, we show that hyperglycaemic conditions reduce miR-222 expression on EC-derived microparticles. Interestingly, this is in accordance with findings from Togliatto *et al*., who demonstrated that hyperglycaemic conditions inhibit EC proliferation through down-regulating miR-222 expression on ECs 37.

On a molecular level, our results complement findings from Rautou *et al*., who demonstrated that the effect of human MP depends on the stage of disease progression in patients with atherosclerosis and liver cirrhosis 38,39. Recently, we showed that iEMP promote ICAM-1 and VCAM-1 expression in resting endothelial target cells 34. In this study, we found that under pro-inflammatory conditions, iEMP rather reduce ICAM-1 expression in target ECs. These at first sight contradictory results suggest that the effect of MPs and probably of miR-222 depends not only on the state of the releasing cells but also on the pro- or anti-inflammatory condition of the recipient cell.

In conclusion, we show that EMP reduce ICAM-1 expression in a miR-222-dependent mechanism in endothelial target cells. Endothelial microparticles derived from high glucose-damaged ECs contain less miR-222 and lose their anti-inflammatory capacity. These results add new aspects to the role and function of EMP as a potential messenger in intercellular communication depending on the functional state of the releasing cell. However, the role of EMP as carrier and protection device for miR is new and some important questions remain and will be addressed in future studies. First, although we see a specific effect of miR-222 in EMP, we cannot rule out the influence of other miR or bioactive molecules present in EMP in our findings. Furthermore, isolation and investigation of circulating EMP from patients with and without diabetes concerning their anti-inflammatory potential is necessary to understand the pathophysiological relevance of EMP in human.

## Funding

NW and FJ were supported by Deutsche Forschungsgemeinschaft (WE 4139/8-1). FJ, KB and DP were supported by the Medical Faculty of the Rheinische Friedrich-Wilhelms-University Bonn (BONFOR). FJ was additionally supported by the “Familie Schambach” Foundation.

## Conflicts of interest

None.

## Supporting Information

**Figure S1** VCAM-1 expression on EMP target cells. **Figure S2** EMP are taken up by target endothelial cells in a time-dependent manner. **Figure S3** ApoE−/− mice, 10- to 12-week old, receiving cholesterol-rich high-fat diet were treated twice per week with 1 × 10^7^ AnnV+ EMP or vehicle over a period of 8 weeks. A) VCAM-1 mRNA levels were analysed by real-time PCR. 18s was used as endogenous control, *P* > 0.05 *n* = 6–8. **Figure S4** Efficient down-regulation of miR-222 in EMP^miR-222-down-regulated^.
