# Supplementary material for: Endothelial microparticles reduce ICAM-1 expression in a microRNA-222-dependent mechanism
Source: J Cell Mol Med. 2015 Jun 17;19(9):2202–14. doi: 10.1111/jcmm.12607 (PMC4568925; doi:10.1111/jcmm.12607)

Supplemental figure 1

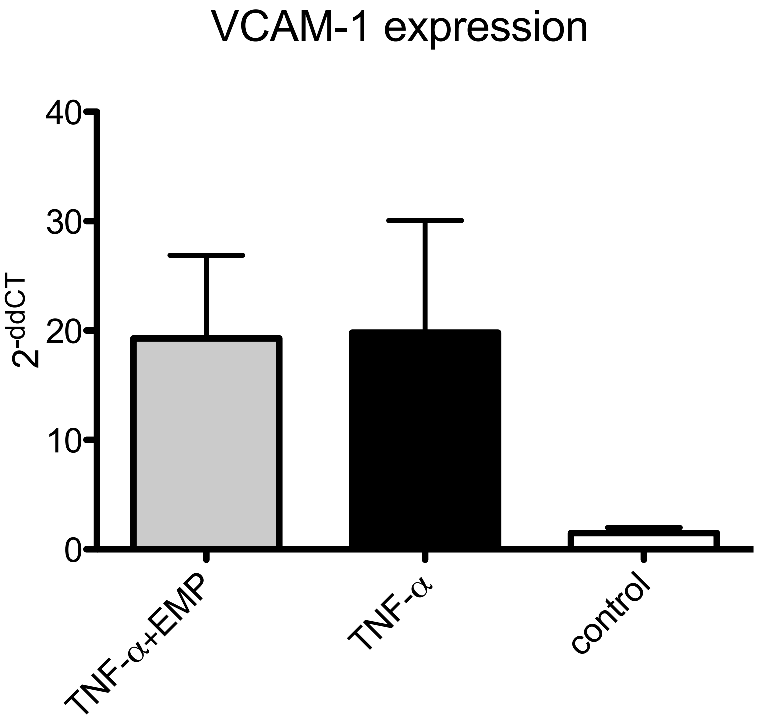

Supplemental figure 2

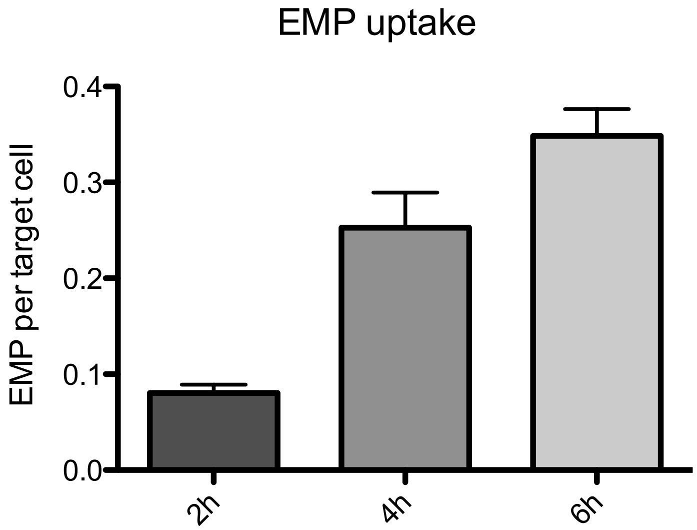

Supplemental figure 3

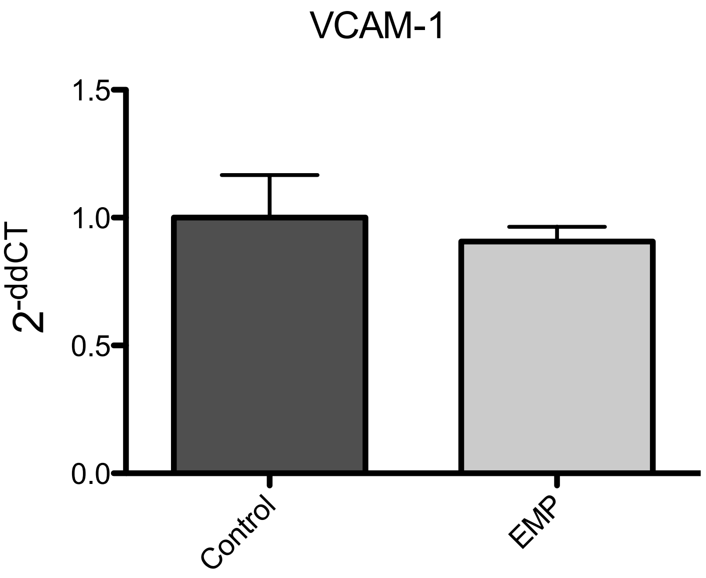

Supplemental figure 4

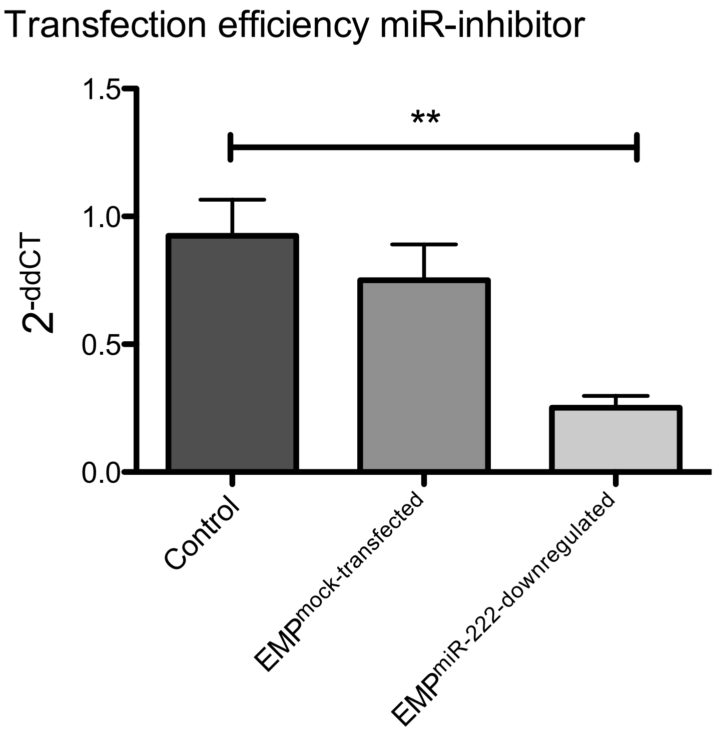

Supplement: Supplementary file 1 [file jcmm0019-2202-sd1.pdf]
